# Are Health Nudges (Dis)Empowering?

**DOI:** 10.1007/s11019-025-10322-2

**Published:** 2026-01-13

**Authors:** Per-Anders Tengland

**Affiliations:** Health and Society, Malmö University, Jan Waldenströms gata 25, 205 06 Malmö, Sweden

**Keywords:** Autonomy, Empowerment, Health promotion, Libertarian paternalism, Nudges, Public health

## Abstract

Health nudges have become popular in public health and health promotion, not least in countries that have liberal or libertarian policies. One reason is that nudges aim to change people’s health-related behavior in a positive direction without infringing upon their freedom. There are other strategies to try to achieve health. Comparing behavior change and empowerment models, previous research found that empowerment is morally recommendable, both as a strategy and as a goal. Empowered people are good at taking care of their health. Using empowerment as an evaluative model, this paper investigates to what extent nudging as a health-promotion strategy leads to empowerment. Nudging can be empowering insofar as it leads to better health, although its effects on people’s empowerment might be rather small, depending on whether the nudge creates a healthy habit or not. However, nudges do not, in general, have positive effects on other empowering factors, such as a person’s knowledge, skills, self-confidence, self-esteem, or autonomy, since nudges typically bypass deliberate reflection. Moreover, nudging belongs to a group of manipulative strategies that appear to be detrimental to people’s ability for autonomy. Thus, nudges risk being disempowering.

## Introduction

The idea of “nudges” became well-known with the book *Nudges—Improving Decisions About Health*,* Wealth*,* and Happiness* by Richard Thaler and Cass Sunstein (2008).^1^ In their book, it is argued that, from a liberal perspective, the strength of a nudging approach is that it non-coercively influences people to make certain choices, while the freedom to refrain from adopting the suggested choice is retained. Thaler and Sunstein do not only focus on health, but also on wealth and happiness (as is clear from the title of their book). Nudges can, thus, be applied to a variety of social problems. But for the purpose of this article, I will primarily discuss health nudges.

Nudges are considered useful in certain areas of health promotion and public health, and can be seen as belonging to a kind of interventions that Andrew Sims and Thomas Müller call “behavioural public policy” (2019, 195)—an “*evidence-based* approach” (205) that uses knowledge from “cognitive psychology and behavioral economics in order to achieve its aims” (195). In doing so, the approach utilizes knowledge about the psychological shortcomings and biases that human beings often exhibit, and works through “targeted interventions into the human cognitive system” (205).

Nudges, and behavioral public policy in general, constitute an alternative to more traditional coercive strategies for health-related “behavior change,” typical of paternalistically inclined policies, such as using legislation or taxation in order for people to make the “right choices.” They are also an alternative to strategies where people are only informed about—or, at the most, persuaded or incentivized to make—healthy choices, that is, less paternalist strategies, typical of more liberal and conservative policies (Downie et al. 1996; Glanz et al. 2008; Baum 2016). The strategy of using health nudges is paternalist in that it (necessarily) aims at benefitting the person affected, disregarding what the person wants. However, it is still libertarian, since it supposedly retains freedom of choice (Thaler and Sunstein 2008).

There is, however, yet another alternative approach to health promotion, namely, an empowerment approach. Such an approach aims at strengthening people’s abilities and skills as much as possible, as well as their ability for autonomy,^2^ positing that people with (at least) a reasonable control over their circumstances are able to live healthier and better lives (Laverack 2016; Baum 2016, 524–544). In two previous papers comparing behavior-change models and empowerment, I argued that empowerment is both the more important goal and a more effective strategy, and—as both a goal and a strategy—ethically more sound (Tengland 2012; 2016),^3^ without, however, discussing nudges.^4^ More about empowerment later.

Scrutinizing the literature on health nudges, there is little (explicitly) said about nudges’ relation to empowerment, although quite a lot is written about the right to autonomy, a key aspect of empowerment. A few articles, however, present alternatives to nudging, empowerment being one such alternative. Goodwin (2012) and Mark Button, (2019) both contrast empowerment with nudges. Neither of them presents a precise definition of the concept, but Button in particular seems to consider empowerment to be a deliberative ability, more or less like autonomy. In their discussion of “boosting,” as an alternative to nudging, Ralph Hertwig and Till Grüne-Yanoff (2017) clearly have something like empowerment in mind, even if they use the term sparingly. Similarly, Peter John, Graham Smith, and Gerry Stoker’s (2009) proposed goal to make people “think” is obviously about empowerment, but that term does not appear in their text.^5^ Interesting as these articles may be, the specific relation between health nudges and empowerment is still in need of a thorough critical investigation.

The aim of this paper is thus to investigate to what extent nudging as a health-promotion strategy can be expected to make people more empowered. But first we need to better understand what nudges are and also why empowerment can be considered a valid goal for health promotion practice. Since I will be discussing *health* nudges, I will briefly consider what a theory of health could look like. This will provide a more precise sense of what nudging, empowerment, and other health interventions ultimately want to achieve. I will end the paper with a discussion of whether nudges might be disempowering.

## Defining nudges

How to define the concept of “nudges” has been much debated since Thaler and Sunstein’s book was published in 2008. The authors themselves do not provide a strict definition. They do, however, describe a nudge as “any aspect of the choice architecture that alters people’s behavior in a predictable way without forbidding or significantly changing their economic incentives” (2008, 6).

To understand nudges, one theoretical assumption is crucial, namely, that we make choices on two mental “levels.” Kahneman (2011), for example, differentiates between cognitive processes that are *fast and automatic* (system 1), and those that are *slow and reflecting* (system 2).^6^ The latter level is where we deliberate and make autonomous choices. The point is that we cannot deliberate, that is, use system 2, all the time, since it is too time-consuming, so we mostly rely on quicker and “shallow cognitive processes” (Saghai 2013, 489) in making everyday choices (see Kahneman 2011). This is what nudges take advantage of.

Thaler and Sunstein’s description of nudges has some drawbacks, primarily in that it includes too many different health promotion strategies, thereby not clearly separating those that target people’s automatic (system 1) cognitive processes from those that engage the person’s critical deliberation (system 2).^7^ For example, in a later paper, Sunstein (2010) includes examples of strategies that use information and rational persuasion. Other writers follow Thaler and Sunstein in this respect, for example, Robert Baldwin, who categorizes nudges into four kinds, namely, information, warning, default, and design (2014, 10),^8^ and Luc Bovens, who uses “social advertisement” as an example of nudges (2009, 208). And in a scoping review on nudging in public policy and public management, Van Deun et al. find that the most common type of nudges (33%) is “provision of information” (2018, 15).

Blurring the distinction between strategies that work via (more or less) unconscious influences and those that work via conscious ones, is not theoretically satisfactory. It makes sense to differentiate nudges, not only from empowerment strategies, but also from “ordinary” behavior change strategies, such as providing information or incentives. Moreover, Thaler and Sunstein do not differentiate between paternalistic nudging and nudging for other reasons. This is strange, given that they also refer to their position as “*libertarian paternalism*” (2008, 5; italics in the original). I will suggest a definition of nudges that is a bit narrower and more precise.

Note that there is nothing “natural” about the category of nudges, and there is no self-evident way to define the concept. What a nudge is, is a “social construction.”^9^ Usefulness—practical, theoretical, and ethical—is at least one important criterion for a successful definition. For the purpose of this paper, nudges have to be sufficiently different from other health-promoting strategies, such as information strategies, rational persuasion, and coercion, in order to be theoretically interesting. Thus, I will focus on some key features that I (and others) find important, and that make the phenomenon sufficiently unique, and then suggest a definition that seems useful.

Nudges are, as all health-promotion strategies, intentional (Nordenfelt 1991; Saghai 2013; Hill in Wilkinson 2013; Quigley 2014). Since we are dealing with health promotion, and professional practice, I will, as already mentioned, assume that nudges are paternalist. Thus, they have to be intended to result in something positive for the nudged person, in our case health, or some health-related state. Finally, as with health promotion strategies in general, success is not required for something to be a nudge. On the contrary, for nudges the possibility of failure is a necessary condition.

In order to differentiate nudges from health information (social advertising) and rational persuasion, several authors (as we have already seen) stress the fact that nudges in general bypass the nudgee’s conscious, rational, and deliberate thinking. Yashar Saghai, for example, suggests that the choice architecture that makes up nudges only “triggers” (makes use of) people’s “shallow cognitive processes,” that is, processes that are fast, require few mental resources, and do not need full-blown deliberation (Saghai 2013, 489). Saghai, moreover, requires that, in order to qualify as a libertarian strategy, nudges should be easy to resist, that is, be “substantially non-controlling” (ibid.).^10^ He also requires that the set of choices available is not reduced, although it could be expanded (ibid., 488).

Thus, to nudge someone is (a) to intentionally try to influence someone else’s (non)actions, (b) (typically) via the person’s shallow cognitive processes, that is, processes that the person herself is not fully aware of, but (c) where this influence does not substantially determine the actions of the person (that is, leaves her free to easily refrain from acting according to the nudge), and (d) where options are not reduced, – all of this in order to (e) benefit the person. For a more formal definition, I will use Saghai’s, slightly revised:

A nudges B when A [intentionally] makes it more likely that B will φ [phi] primarily by triggering B’s shallow cognitive processes, while A’s influence preserves B’s choice-set and is substantially non-controlling (that is, preserves B’s freedom of choice) [for the sake of B] (2013, 491).

###  Examples of nudges

Some examples of nudges are called for. One such example is about how to organize a cafeteria (Saghai 2013), a self-serving restaurant, or a grocery store, that is, what default display to choose. Depending on where you place the goods in a store people will change their purchasing behavior. And in canteens serving lunch, the healthy food can be placed at the beginning of the food queue and at eye level, and the less healthy foods can be placed further down the queue and be less visible (Thaler et al. 2010). Google used a similar strategy when they put the water bottles at eye level and the soda at the bottom of their fridges, in order to promote a healthier consumption (Engelen 2019, 49).^11^

A further strategy implemented in some countries is to mandate restaurants, and similar establishments, to specify the amount of calories that each meal contains, in order to influence people to choose the “healthier” (low calorie) options.^12^ Such a law has been in place in the USA since 2018 (FDA 2024) and the UK followed suit in 2022 (Gov.uk 2024).^13^ Similarly, some countries require food manufacturers to label their food with different colors, where, for instance, the color green indicates that the food is healthy, red indicating unhealthy food.^14^^,^^15^ This kind of (attempt at a) “subliminal” influence is, as we saw (footnote 8), also found in some “warnings,” such as requiring manufacturers to use unappealing colors, or pictures of “sick” organs, on their cigarette packages (Lang 2016). There is another interesting nudge from the world of dining. Knowing how people read menus, restaurant owners can “plan” or “compose” their menus in order to influence customers’ choices, for example, place dishes with a higher economic margin where people tend to look the most.^16^

Saghai (2013, 487) also mentions a strategy where information is posted at a university campus about how much alcohol students typically drink each week, in order to make them understand that other students drink less than they imagine. The belief is that students will (binge) drink less when they know that other students do not (binge) drink that much. Similar social norms can be used in other contexts, for example, displaying on the office bulletin board how much people in the company in general exercise.

A number of examples describe ways to encourage more physical activity.^17^ Thus, cities can be made friendlier to both walk and bike in, for example, by building more bike lanes, while preserving all other transportation options. You can also change the environment by creating parks and other recreational areas in order to nudge people into exercising more. Furthermore, when planning the construction of new buildings, architects can make the staircases available and inviting, so as to encourage walking the stairs, and place the elevators or escalators further away from the entrance, or make them less conspicuous. Another way is to “gamify” the environment (Engelen 2019, 49). One such strategy is to try to make people take the stairs by making them interactive, for example, in the form of a piano keyboard, where the steps play tones when you walk on them, thus encouraging playful walking.^18^ Today people are also inspired to walk more by having a health app with a step counter in their cell phones.

Two final kinds of nudges should be mentioned. First, there is the kind called *framing*, which alludes to the fact that how a person, for example a health promoter, frames the available choices partly determines the choice of the person (Thaler and Sunstein 2008, 36). The same health information can be framed in different ways. A positive example can be used, by saying that the risk of colon cancer may be reduced, and longevity increased, if more vegetables and fruit are consumed. Or health information can be framed negatively, by stating that the risk of colon cancer, and premature death, increases if you do not eat vegetables and fruit.

Another kind of nudge is called *defaults* (ibid. 83–87) and concerns having an opt-in or an opt-out clause for specific choices, for instance, regarding whether your organs could be used for transplants, should you suddenly die. This, however, is not a health nudge, but the example above about how to arrange the food items in a canteen (or store), in order to promote healthy eating, belongs to the default category.

Nudges have been quite common in health promotion and, like many paternalist actions, they appear to be morally approvable and recommendable. However, nudges have also been questioned on various grounds. They are said to ignore autonomy/freedom, reduce responsibility in decision-making, lack transparency, be deceptive and manipulative, and even be coercive (White 2011, 2013, 91; Quigley 2014, 142; Caloia 2015; Kuyer and Gordijn 2023). These critiques will only be dealt with here insofar as they are relevant for the purpose of the paper.

## The goals and values of health promotion

Health—though not the highest prudential good, which is, or so I will assume, well-being (quality of life, the good life^19^) and living a meaningful life—is the obvious goal for health promotion. But being able to influence someone’s health directly is difficult in health promotion (Glanz et al. 2008, p. 16; Baum 2008, 460 ff.; Tengland 2012), since professionals do not have direct “access” to people’s bodies and minds.^20^ So it has to be done indirectly, via some kind of instrumental goal. In two previous papers I compared and discussed two candidates, namely, as mentioned above, behavior change and empowerment (Tengland 2012, 2016), where behavior change interventions specifically aim to change people’s health-related behavior in a predetermined, positive direction.

I argued that on ethical, and partly empirical grounds, empowerment, both as a goal and as a strategy, was to prefer. I also argued that empowered people are good at taking care of themselves, and their health.^21^ This is rather obvious if we look at, for example, the extensive research done by Marmot (2004) and colleagues (Commission on the Social Determinants of Health 2008). The social gradient clearly shows that more empowered people have better health and a longer lifespan. That is, those who have more resources, both internal and external, for example, skills and confidence, as well as financial, social, and cultural capital, tend to be healthier and live longer. To conclude, empowerment appears to be the most reasonable aim for health promotion.

## Empowerment and health

In the following sections, I will introduce the concept of empowerment, followed by a brief presentation of what I mean by health.

### Empowerment

In scrutinizing health nudges, I will here use (a theory of) empowerment as an evaluative tool. Empowerment has two aspects (Tengland 2008). First, it is a dynamic condition or state of being (and a goal to be achieved),^22^ and being empowered as a state (and a goal) means that the person is well-equipped to control the determinants of their own well-being.^23^ Second, empowerment is a process, which refers to how professionals work with citizens, users, or patients (Wallerstein 1992; Baum 2016, 524 ff.; Laverack 2016, 3; Haapanen et al. 2024), often using a participatory approach. This participatory approach will not be discussed more, but will function as an example of how to achieve empowerment as a state.

Since being empowered is by definition related to well-being, it has great instrumental value for the person. Here is a definition of empowerment as a (dimensional and dynamic) condition, or state of being:

A (any) change (internal or external to the person) is an increase (decrease) in empowerment if (and only if) it is an increase (decrease) in the person’s control over the determinants of her well-being (adapted from Tengland 2008, 90, and abbreviated).

Some key factors that, in general, contribute to the (increase in) a person’s control over the determinants of well-being are: (increases in) health (especially mental health), various kinds of knowledge and skills (not least occupational), self-confidence and self-esteem, and a supportive external environment (that allows a rich variety of actions and activities), as well as social and financial opportunities. Finally, (the ability for) autonomy is crucial for empowerment, since the ability for autonomy is necessary for the person to choose their life course. This importance will be high-lighted in the discussion.

###  Health

Health, I suggest, is a pluralistic notion. First, we have to differentiate between manifest and fundamental health, and, second, on the manifest level, between health as ability and health as “subjective well-being” (Tengland 2007, 2015).^24^

Manifest^25^ health is a holistic feature of the person, whereas fundamental health is a reductionist one. Fundamental health consists of those (non-conscious) states and processes in the person’s mind and body, primarily her anatomy, physiology, and neurology, that support or uphold the manifest health of the person, namely, her ability and subjective well-being.^26^

Ability should also be interpreted pluralistically. It refers to all of a person’s *basic* abilities and dispositions, that is, those abilities that we as children typically develop without any special teaching or training, for example, the abilities to stand, walk, see, hear, grab, chew, swallow, think, talk, and remember,^27^ and the dispositions to experience emotions, to fall asleep when tired, and to be motivated, that people typically develop. Manifest health also encompasses the second order ability to acquire competences (see Tengland 2007 for more details). Not only should all basic abilities and dispositions have been acquired, the healthy person should also be able to use them here and now, assuming that the environment permits such use (Nordenfelt 1995, 48). “Subjective well-being” (how you feel), the other aspect of manifest health, refers to those *sensations* and *moods* that have an immediate internal cause, for example, feeling fit and experiencing joy (for no specific external reason), and, on the negative side, experiencing pain, or anxiety.^28^

Thus, if health promotion succeeds, for example, through inspiring people to exercise more, this means that they typically increase their fundamental health, say, the strength of their heart, or their lung capacity, which in turn affects their manifest health, for example, making them *feel* fitter and/or *be able* to walk or run longer distances. And if people are influenced to eat well, all the body’s organs, not least the brain, will be provided with the nutrients they need, which will, in turn, provide support for all (manifest) conscious activity and action, such as solving problems.

## Discussion

The discussion aims at determining to what extent nudging as a method for health promotion achieves empowerment as a state (of being). Thus, the question is: *do health nudges contribute to empowerment?* I will start by discussing some of those factors where empowerment effects seem least likely, then turn to factors where positive effects seem possible and, finally, discuss the most important issue, namely, autonomy.

### Does nudging lead to increased empowerment?

It is difficult to see that nudges contribute to people’s self-confidence and self-esteem. How do we acquire these mental states? Confidence, whether it is general or task- or situation-specific, is a kind of belief about one’s competence(s), one that is usually gained through succeeding with various kinds of actions and interactions. Self-esteem—which I take to be more of an emotion than a belief—has to do with how one perceives one’s value as a person (such as being satisfied with who one is, or liking oneself), and is primarily acquired in healthy communication with people that one is close to, such as parents, partners, and friends (Illouz 2012, 151). Even if communication processes that lead to self-esteem are not always fully understood, they seem to be of a very different kind compared to the “triggering” of “shallow thought processes” that lead to unreflective behaviors. Thus, since the successful actions that normally lead to self-confidence, and the kind of communication required for self-esteem, are so different compared to the unconscious, or semi-conscious, effects of nudges, it is unlikely that they can be attained through nudging.^29^

Neither do nudges appear to lead to the acquisition of new knowledge or skills. This is, once again, mainly because there is not in general any awareness of the nudge—a prerequisite for knowledge and skills—but also because no (real or reflective) action is taken that you can learn from. There is, however, the idea that nudges could, or even should (for ethical reasons), be transparent, that is, that people that are nudged should be told about them (see Schmidt 2017) and, therefore, become aware of them. Still, it is not likely that much is learned that strengthens empowerment. Some of the knowledge that could be gained is often already relatively well-known, for example, that vegetables are good for you, that sugary drinks are bad for you, or that exercise contributes to your health. But that might be a false assumption. It appears that some groups lack the awareness of things that others are familiar with. For example, research shows that people often underestimate the amount of sugar in various kinds of drinks and foods (Ramji et al. 2020), one factor that tends to lead to overweight. But this is not an argument for nudging; it is rather an argument for better education and health literacy.

Can people’s empowerment be increased through nudges such as the creation of new (health-related) opportunities? Let us assume that the municipality constructs an easily accessible recreation area just outside of the city border, in order to promote the health of the citizens —one that becomes popular and where people, for example, walk, jog, practice tai chi, or cycle. The area might in itself increase well-being, since it is enjoyable for some just to be there, but it is also possible that the citizens’ health improves as a result of their activities in the recreation area, and therefore also their empowerment, at least to some extent. However, besides improved health, very little else seems to increase empowerment in this scenario.

Assuming, as implied above, that nudges increase health, there is an obvious connection to empowerment, since health, in general, and mental health in particular, contributes to a person’s empowerment through making various kinds of action easier or possible. But is this only a small contribution to empowerment, or can such a change make a substantial difference? Before developing this theme more, let us start with the contrary assumption. Schramme (2024) is skeptical of nudges, and argues that nudges can in fact *reduce* health.^30^ People in general, he claims, resent being nudged and this resentment will (supposedly, through physiological processes) eventually lead to an “increased risk of health impairments” (ibid. 33), that is, “depression or heart disease” (ibid. 42). If Schramme’s reasoning is correct, it is difficult to see that health nudges can be empowering.

I doubt that his argument is tenable, however. One problem with the argument is that if nudging works, as Saghai suggests, through shallow cognitive processes, then people would in general be unaware of most nudges, and they cannot resent what they do not know. Moreover, it seems implausible for anyone to be resentful (enough to become ill) if they realize that the fruit has been moved closer to the checkout (instead of the candy), that the stairs in the newly constructed office building have been made more accessible than the elevators, that bottled water has been put at eye level in the fridge instead of the soda, or that the menu states the calories for each meal (see Engelen 2019 for similar reasoning). The one thing that might give rise to resentment, I imagine, is if a person’s favorite food has a red warning label on it stating that it is not good for you.

As to people’s reactions to nudges, contrary to Schramme’s suggestion, some studies found that people, when asked to evaluate the use of various hypothetical kinds of nudges, often approved of them (Sunstein 2015; Reisch et al. 2017; Evers et al. 2018; Krisam et al. 2021). There have, however, been claims that people are more reluctant to accept government nudges than nudges from, for example, experts (Evers et al. 2018).^31^ If this is true, there could be some support for Schramme’s idea. However, a person who might resent being nudged should at the same time realize that there is seldom any neutral “choice architecture,” so that, mostly, a choice is made by someone, often the commercial market, and why would the person nudged not resent these other suggested choices as well, risking depression or heart disease in the future? Furthermore, the person is always (substantially) free to ignore the nudge, a fact that should soothe the (presumed) resentment.

So, in what way could health nudges improve health? Let us assume that placing the fruits closer to the store’s checkout in fact leads to more fruit consumption. The short-term effects seem to be negligible, since very little happens to the manifest health of the (healthy) person that eats an extra apple.^32^ It may, of course, have some minuscule positive effect on the person’s physiology, that is, their fundamental health. But, in order for the nudge to make a real difference we have to assume that it starts a long-term habit.

It would be different if the person suffered from malnutrition, for example, had a mineral or vitamin deficiency. In such cases nudges could make a substantive difference in both fundamental and manifest health, even in a short time span. But it seems that what people need in such situations is information, not nudges.^33^ More generally, in an environment where “junk food” is the standard option,^34^ nudging the citizens into eating more vegetables and fruit could (over time) have a considerable positive impact on both kinds of health (in that the nutrients’ positive effects on physiology would result in people feeling better and becoming fitter), and simultaneously reduce the risk of future disease. However, providing opportunities to buy healthy food, and at affordable prices, is, of course, even more important, and a precondition for such nudges.

As already indicated, it usually takes time for changed eating habits to have effects on health. This primarily has to do with the fact that a person’s manifest health is reliant on their fundamental health, and fundamental health mostly changes slowly, as when a regular increase in the intake of vegetables slowly improves the gut microbiota, which eventually contributes to improved brain functioning and mood (the so-called gut-brain axis; Di Napoli et al. 2024).^35^

What might have a greater immediate effect on health, is, for example, when someone is nudged to start exercising, for example, by taking a brisk walk in the park, and develops a habit to do so.^36^ This will normally strengthen the person’s heart and muscles, increase lung capacity, and, moreover, improve mental health, as well as the person’s mood (Hansen 2016). Thus, to the extent that health nudges succeed, and have health effects, they contribute to people’s empowerment, since health is in general important for most things we (want to) do, such as working, socializing, and playing.

Finally, let me turn to the ability for autonomy, which might be seen as consisting of several related aspects: the ability to reflect upon and determine one’s higher-order values and preferences, to deliberate critically on choices of how to act, and, finally, to act on choices made,^37^ provided there is an external opportunity to do so (freedom).^38^ Autonomy is crucial, since without the ability for self-determination there would be no empowerment, that is, without autonomy reflective choices about one’s conception of the good (one’s well-being) cannot be made. Some bodily or mental conditions can have a considerable effect on the autonomy of individuals, such as dementia, drug addiction, congenital cognitive impairments, or severe mental disorder. But since there are degrees of autonomy, it can also be reduced to a lesser extent.

Now, it seems clear that nudges do not contribute to a person’s (ability for) autonomy, since the person nudged is supposedly unaware of the fact, and since autonomy by definition, as we saw, requires conscious deliberation. But, once again, what if the authorities are transparent with their nudges, as Schmidt (2017) suggests? Then the conclusion is not as self-evident. Perhaps if you become aware of the fact that you are nudged, you might reflect critically about why, and about the choice suggested, and, therefore, use and develop your autonomy. But this will only be an accidental byproduct of the nudge. So, why nudge in the first place, and not just use some other strategy? And then, again, we might ask if this really is nudging.

Thomas Nys and Bart Engelen (2015) have an argument that purports to show that nudging might increase rationality, and, by implication, autonomy. They reason as follows: A choice architecture can, through non-rational influences (a nudge), “help us do what we consider best [for us]” (2015, 205). Our rationality (to get what is best for us; buy fruit, not the candy) might sometimes require “the use of mechanisms that do not operate *via* rationality” (2015, 205; italics in the original). Thus, “bypassing [our rational capacities] can be required exactly to *enhance* our rationality” (205; my italics). True, the nudged person gets what they rationally want, but this does not show that nudges *enhance* the chooser’s rationality. Moreover, it does not even seem *rational* to just get what one considers best, if it is not achieved through deliberate choice. Furthermore, an increase in the ability for autonomy (of which rationality is part, or a precondition), which is the important issue here, is unlikely to come from nudges, even if we get what we want, since autonomy does not just happen. The person *has to do* something intellectually for it to develop.

The conclusion of this section must be that nudges, despite having some positive health effects, have a relatively moderate causal impact on people’s control over the determinants of their well-being, that is, their empowerment.

### Can nudges undermine a person’s ability for autonomy and be disempowering?

A number of authors claim that nudges are problematic since they disrespect *the right to* autonomy (for examples, see Kuyer and Gordijn 2023, 198 ff.). That might be true, but there is a greater worry: could nudges instead even *reduce the ability for autonomy*? It can be argued that it could. However, even if some of the critical literature on nudges has raised such concerns, it presents relatively few arguments. Therefore, finding good arguments that support the claim requires a brief detour.

In his book *Stand out of our light: freedom and resistance in the attention economy* (2018), James Williams describes and discusses what he calls the “attention economy.” Historically, humans have lived in “environments of information scarcity” (ibid., 13), but with a great need and desire for it. Today, there is an abundance of information (for example, the internet), but “when information becomes abundant, *attention* becomes the scarce resource” (Simon in Williams 2018, 13). Given this situation, the major digital tech companies have devised strategies, primarily to trigger the brain’s different reward systems, in order to manipulate people to spend as much time on their platforms (looking, reading, listening, clicking, scrolling, tweeting, liking, etc.) as possible. These strategies are to a large degree based on the psychology of human cognitive biases (see, for example, Kahneman 2011).^39^ The reason for using these strategies is that the tech companies make money by selling users’ attention to commercial companies that want to expose their goods and services. The major tech companies have collected massive amounts of behavioral data about their users, which means that the companies (manufacturers and advertisers) that buy these data have specific knowledge about their potential customers—knowledge they can use to target individuals who might fancy their products. Shoshana Zuboff (2019) calls this “surveillance capitalism.”

Moreover, companies strive to create new needs among their viewers and customers, some of which are acquired, but often not autonomously reflected upon and, thus, not “authentic,” that is, do not really contribute to well-being. And the companies in question succeed in attracting people’s attention: An average American spends 5.4 h a day on their smartphones (Hari 2022, 76) and recently, a Swedish newspaper (Sydsvenskan: June 2024) reported that Swedish youth spend on average six hours a day on their smartphones. It seems unlikely that people spend these hours choosing activities on their phones, or other digital devices, entirely autonomously. It is rather, I maintain, Kahneman’s shallow system 1 that takes over.

The constant bombardment of stimuli and manipulation, Williams argues, *reduces the users’ ability for autonomy* (2018, Chap. 9). In this ceaseless flow of “information,” mixed with commercialized ideals and values, there is little time for the person to step back and rationally reflect on their choices and “the good life.”^40^ Finally, many people, not least youth, appear to be psychologically “addicted” to these platforms, and would likely have a hard time refraining from using them even if they tried (see Zuboff 2019, 449–452), which is, of course, the antithesis of autonomy, and therefore also of empowerment. That such processes can occur is vindicated by psychology. Brains are plastic, that is, adaptable, and when a person is constantly nudged or prodded, there is a risk that they may increasingly stop deliberating over their choices. Once a neural pathway has been established, “moving out of this too-well-ploughed furrow can be very difficult” (Rebonato 2013, 47).

The connection between the above reasoning and nudges should be clear. Nudges too are, as we have seen, “manipulative” (using people’s shallow cognitive processes) and bypass deliberation. In fact, many of the strategies on the web resemble nudges. The worry, then, is that health nudges in a similar way, as with the attention economy, will reduce people’s ability for autonomy. Several authors have voiced such concerns. Nudges undermine the nudgee’s “control over his or her ability to choose,” claims Goodwin (2012, 89), as well as their agency (Hausman and Welch 2010). Bart Engelen adds the risk of perverting the person’s ability for rationality in decision-making (2019, 54). In their systematic review of ethical issues concerning nudging, Paul Kuyer and Bert Gordijn (2023) provide similar claims from several writers. They find claims to the effect that nudges “corrupt people’s higher-order preferences” (ibid. 203), as they “interfere with an agent’s ability to choose her own ends” (ibid. 203). Robert Baldwin is worried that nudges “may involve re-shaping the target’s idea of their own welfare” (2014, 14). They might also lead to a “fragmented self,” claims Bovens (2009, 217), in that successful nudges could result in the person perhaps “no longer recogniz[ing] her choices as her own” (Kuyer and Gordijn 2023, 203). Similarly, people might come to question their own true preferences and even start to doubt their ability to form proper judgements (Dold and Schubert [2018] in ibid., 2023).

But how serious is this critical position regarding nudging in general, and its effects on autonomy in particular? It appears to me that it has to do with the extent of nudging and manipulation. Nys and Engelen’s (2015, 206) division of types of (possible) nudgees is useful; they divide them into those who (1) already have a higher-order preference for the suggested choice in question (buy fruit, walk the stairs), (2) have a reflective (second-order; system 2) aversion against the suggested choice (dislike fruit, hate walking the stairs), and (3) do not have a clear preference. It seems that it is the last type of nudgee that primarily merits moral consideration. The first type is only reminded about what they already (autonomously) prefer, and the second will resist any attempt to nudge them, which should be easy, according to Saghai’s version of a paternalist nudge. It is primarily the undecided whose autonomy (in every specific case) will be at risk. On the other hand, each person can be in all three categories at the same time, though related to different kinds of nudges, so few of us are totally unaffected by nudges.

Despite the concerns raised above, a nudge now and then can seem rather inoffensive, and each case might appear morally acceptable on its own. But several authors primarily worry about the effects of *long-term nudging*, that is, “multiple instances of nudging” over time, on autonomy, what Kuyer and Gordijn categorize as “infantilization” (2023, 214–215), a term borrowed from Bovens (2009). Thus, according to some authors, nudging might lead to: reduced stimulation to make one’s own decisions (Rebonato 2013); less experimentation in choices (Schubert 2017); the acceptance of faulty preferences (Binder 2014, 524); a distortion of the process that takes place when one deliberates about one’s own preferences, a process which also has intrinsic value (Dold and Schubert 2018), and not being fully able to learn from the process; the impairment of the “ability to improve morally” (Burgess 2012); the risk of “forego[ing] the chance to gain the virtue of self-command” (Bovens 2009, 218); and the risk that individuals will “learn to be dependent and inactive” (Binder and Lades 2015, 17).^41^ All of these statements relate to autonomy, in one way or another, and, thus, at the same time to empowerment. Baldwin summarizes this well in his fear that people’s “autonomy [..] dies the death of a thousand nudges” (2014, 14).^42^

Taken together with the already massive manipulative commercial strategies – of which the surveillance and attention economy is one important aspect, aimed at making people think less, rather than more, and who therefore risk losing their deliberative ability—nudging as a strategy to promote empowerment, seems to be a rather bad choice. Some claim, however, that a choice architecture is mostly already present, so that we cannot get rid of the “problem.” It is better, then, to nudge paternalistically, when possible, than let some commercial agents create the choice architecture, or leave it to chance (Nys and Engelen 2015). Whether we should accept that claim or not, will differ depending on the kinds of nudges. Arranging how to display the food in the grocery store or a self-serving canteen (defaults), making either the stairs or the elevators most accessible in a new building, and framing certain choice situations – these are situations where choices seem to be necessary and where paternalist nudging can be seen as appropriate. But other nudges, that are not necessary, can be seen as less appropriate, such as labeling foods with colors, posting information about people’s behaviors, gamifying environments, and using warning labels, for example, on cigarette packages.

### Alternative approaches to achieve empowerment

There are, however, many other options available when promoting health – options that truly empower people. Participatory strategies, aimed at knowledge, skills development, consciousness raising, and strengthening confidence, self-esteem, and autonomy, are alternatives to nudging and to behavior-change strategies in general (Tengland 2012; Baum 2016: 534–564; Laverack 2016; Buchanan 2023, 235–249; Haapanen et al. 2024). Hertwig and Grüne-Yanoff (2017) make a similar case in arguing that “boosting” is preferable to nudging. What they call boosts are clearly empowering, since the strategy focuses on fostering “enabling competences.” The same goes for the “think” strategy, which endorses community-based public deliberation and discussion (John et al. 2009), and for the public, deliberative democratic processes that Button suggests and describes (2019). Moreover, Martin Binder and Leonhard Lades suggest what they call Autonomy-Enhancing Paternalism (AEP). Their response to nudging is to find strategies that improve “individuals’ ability to make critically reflected, unbiased, autonomous decisions” (2015, 4). Thus, AEP aims at strengthening the person’s ability for autonomy.^43^ Many of these suggestions overlap with the suggested empowerment approach, although some such initiatives and interventions come from professionals or politicians, and are not participatory.^44^

Most of this would probably not impress the libertarian paternalist of the instrumentalist kind, since “they do not place much emphasis on, or [show] interest in, the importance in learning ‘how to think’” (Rebonato 2013, 19). There is even an implicit objection from the libertarian paternalist to these assumptions, namely, that the existing cognitive biases that are a starting point for the strategy of nudging make certain “teaching” or autonomy-raising projects more or less futile (Rebonato 2013, 43–44). We are assumed to be “hard-wired to be imperfect thinkers” (ibid. 43). But this is questionable, and the assumption has been challenged. Social psychologist Richard Nisbett has shown that rationality and intelligence are teachable, and that even people’s IQ can be improved, thus, also those areas that were previously considered “close-to-immutable” (in Rebonato 2013, 45).

The alternative approaches mentioned above are often more complex, difficult, and costly to implement, but they might be necessary if we want to make real positive change in people’s lives and health. Moreover, utilizing system 2 also requires more time and resources from the person who is deciding (compared to system 1), which might, according to some, be seen as a loss of welfare (Binder and Lades 2015, 17) and risk leading to “decision fatigue” (Besharov in ibid., 18)—a concern that, to me, seems exaggerated. The strategy should be to focus on autonomously deciding on health-related habits and virtues (for instance, deciding to always use your bike helmet, never eat meat, practice moderation when drinking alcohol, etc.) and then stick to them as much as possible. One might, furthermore, expect that the more people practice their ability for autonomy the more effective it becomes, and one might further expect that autonomous choices, despite being a possible short-term burden, are likely to lead to long-term satisfaction or well-being. That is, life is likely to be better lived.

One might, of course, ask how big a problem a supposed reduction of autonomy is, and wonder about the empirical support the worry about such a reduction has. That autonomy is regularly bypassed in nudging strategies does not in itself prove that the ability is reduced. This is one reason for making a comparison with the surveillance and attention economy, which uses similar strategies and where it appears obvious that autonomy is, not only bypassed, but also reduced. Williams’ reasoning about the constant distractions of the internet, and how they crowd out reflective and deliberative thinking, is quite convincing (2018, Chap. 9). Once again research vindicates Williams’ reasoning: “[*N*]*ot* encouraging individuals to make conscious, deliberate, and well-reasoned choices can reinforce the System-1 neural wiring of the brain,” and “the less the neural pathways associated with our reflective mode of thinking are activated/…/[t]he more/…/our brain will *become* ill-equipped to reason rationally and critically about the next choice” (Rebonato 2013, 49; italics in the original).

Moreover, economists George Akerlof and Robert Shiller (2016), having researched another manipulative phenomenon, what they call “phishing for phools” (which is also the title of their book), come to a similar conclusion: we know that people make “mistakes” (in our terms, fail to utilize their second-order deliberative ability) since people “choose” things that “NO ONE COULD POSSIBLY WANT” after deliberation (2016, xii; upper-case letters by the authors). They mention several areas where this regularly happens, for example, people’s personal financial security (buying things they cannot afford); their health (eating and doing things that might kill them); and their voting behavior, that is, voting for candidates that will make their lives worse (ibid. xii-xvi). It is unlikely that nudging is the answer to such problems.

Another, final, observation is clearly worth mentioning. Critical deliberation over values, choices, and actions is a learning opportunity (Binder and Lades 2015), through which a person’s preferences, as well as their identity, can be restructured. This means that every time an opportunity for deliberation is lost because of a nudge (or some other manipulative strategy), “potential” autonomy is reduced—an observation that should be seen in the light of what was written above about the plasticity of the brain.

## Conclusion

The primary aim of this paper was to investigate if health nudges (as a strategy) can be empowering. Insofar as the reasoning I have presented is correct, nudges, if successful, will primarily, for example, through improvements in food consumption, or through exercise, have some positive effects on a person’s health, which will contribute somewhat to empowerment. However, to have substantial effects, from both a health-promotion and an empowerment perspective, the nudges have to be successfully repeated over time, or, preferably, the health-related “behaviors” encouraged by nudges have to develop into habits (or rather, hopefully, into deliberate personal health strategies).

Moreover, some opportunity-increasing health strategies, such as building bike lanes, parks, and other recreational areas, seem to be empowering (at least for some), both directly, since they expand people’s options, and indirectly, through their possible impact on health. It is, however, debatable if all such environmental changes should count as nudges, since their use might require awareness and deliberation.

Possible health benefits apart, nudges are not likely to have many positive effects on a person’s knowledge, skills, self-confidence, or self-esteem (other areas that contribute to empowerment), and neither on autonomy, since deliberate reflection is usually bypassed. If, on the other hand, nudges are made transparent and give rise to reflection, this conclusion might have to be qualified. Then the question is if they can be seen as nudges at all.

Finally, nudges belong to a group of manipulative strategies that may be detrimental to people’s ability for autonomy, and therefore risk being disempowering. When nudging, despite being paternalist, becomes part of our commercial society’s constant, long-term bombardment of manipulative stimuli and “information,” we can seriously question if nudging is the solution, or even part of the solution, to our health problems – especially when there are other, much better, ways to achieve our health promotion goals, such as participatory empowerment strategies.

## Footnotes

1 The idea is not entirely new in a health context. As Vallgårda (2012) has pointed out, similar strategies were found earlier, for example, in Nancy Milio’s suggestion that we “make health-generating choices more easy” (1976, 435). See also WHO’s Ottawa Charter for Health Promotion (1986), and subsequent charters.

2 Since the theory of health presented further on in the paper focuses on people’s abilities, I have used “ability” throughout the paper, rather than “capacity”, a term which I here take to be synonymous with the former.

3 Button (2019) reminds us of the theoretical importance of emphasizing that empowerment, boosting, and similar strategies, are concerned with strengthening agency, i.e., the capacity for action, and not with behavior. He quotes Hannah Arendt, who was critical to the “substitution of behavior for action” (Button 2019, 1037–1038).

4 Note that these kinds of “health strategies” are “ideal types” (Weber 2019[1921], 96–99), and that they might sometimes overlap in real life.

5 Some government officials appear to believe that nudges “‘empower[] individuals to make healthy choices’” (Department of Health, quoted in Nys and Engelen 2015, 201). Button (2019, 1039) notices the contradiction in this kind of government rhetoric.

6 Hertwig and Grüne-Yanoff (2017) contest the idea that there are only two kinds of choosing (system 1 and system 2), claiming that consciousness is more complex than that. Be that as it may, focusing on these two (ideal) types of cognitive processes can still be theoretically useful.

7 Not making this distinction, some seem to think that one can nudge “system 2” (see Haug and Busch 2014). The definition of nudges that will be proposed here makes that impossible (by definition).

8 “Warnings” are of two kinds, one that is manipulative (as nudges are), in that they aim to affect people “subliminally”, that is, without the primary intention to make them think critically about the issue in question, and another kind that tries to evoke awareness and discussion by being disturbing or “scary”.

9 Whatever is categorized as a nudge is nevertheless real (under some description).

10 What Saghai calls “prods” are, on the other hand, intended to be “substantially controlling”, in that they are more difficult or impossible to resist, for example, automatically enrolling people after a try out period. They are not usually paternalist either.

11 Water consumption went up by 47% and soda consumption down by 7% (Engelen 2019, 49).

12 It can be questioned whether this strategy should count as nudging, since it seems to require deliberate thinking, and does not primarily “trigger” people’s shallow cognitive processes. It resembles health information.

13 This obligation only concerns major restaurant chains, those with 20 or more branches.

14 For an example of how this is done, see the Singapore Health Promotion Board (2024).

15 This seems more like nudges than stating the number of calories, since the colors are obviously meant to be “triggers” for certain behaviors.

16 The research evidence is, however, not definitive, so more cannot be said about the strategy here.

17 Note that some of the strategies mentioned might not be self-evident nudges, since, first, they might not be intended as such, and, second, in some cases they might not primarily trigger people’s shallow cognitive processes, but instead inspire people to make deliberate decisions, for example, as when a person because of the construction of new bike-lanes decides to start commuting to work on bike. But I will ignore this complication here.

18 The idea of making the stairs into a keyboard was first tried in a Stockholm subway station (Odenplan), and has since then been tested elsewhere. Watch here: https://ljudplanering.se/2010/04/22/pianotrappan/ The increase in people choosing the stairs was 66%.

19 I will here treat these three terms as synonyms.

20 A few exceptions are: vaccinating people, fortifying foods, and putting fluoride in the drinking water. It is different in medicine where doctors do all sorts of things with people’s bodies in order to heal them. Other professional activities that usually have direct effects on someone’s health are massage, chiropractic, and acupuncture, and, for mental health, psychotherapy.

21 David Buchanan makes a similar claim about autonomy (2023, 205), and, as I will show, autonomy is the most important aspect of empowerment, so our positions are quite close.

22 I will use the word “state” and assume that it covers a variety of states (of being), conditions, and even statuses, such as being married, holding a citizenship, or having a PhD.

23 This applies to groups as well, but for simplicity’s sake I will focus on individuals.

24 This notion is distinct from “well-being” in the quality-of-life sense, as will be made clear further on in the paper.

25 I will use ability and capacity interchangeably.

26 Beside these “functions,” there are a few crucial dispositions found on this “level,” for example, resilience, stress tolerance, emotional stability, control over one’s drives, and ability to recover.

27 The abilities are of two kinds, intentional, such as walking, and automatic (or inborn), such as hearing.

28 Emotions, a third kind of feeling, typically have external upholding causes, and do therefore not constitute health states. See Nordenfelt (1995, 82 ff.) for an elaboration of these distinctions.

29 But consider a possible example where it might be acquired: a person is nudged to walk the stairs, gains a habit to do so, becomes and feels more fit, and therefore becomes more satisfied with themselves. Thanks to Katarina Graah-Hagelbäck for challenging me on this point.

30 In his view, health is either the absence of disease, or “fitness,” meaning, more or less, the propensity to not fall ill (resilience to illness) (Schramme 2024).

31 However, in a German study they found no such differences (Krisam et al., 2021).

32 An exception is to nudge people to take some stimulant, such as a cup of coffee or black tea, in order to make them more alert, but I have never seen such health nudges (except as advertising prods for coffee). People are, however, often prodded to drink sweet beverages, some of which might be “energizing.” Such commercials are, of course, the antithesis of health promotion (since excess sugar is considered bad for your health), even if they “pick you up” temporarily. Most such effects on manifest health are, real as they might be, rather short-lived. See Tengland (2015) for a general discussion of the relation between stimulants and health. These exceptions apart, almost all nudges target people’s fundamental health, not their *immediate* manifest health.

33 It would even seem morally wrong only to nudge them instead of providing them with facts, or food.

34 Some people live in areas or cities where fresh fruits and vegetables are hard to come by. What they need are opportunities, not nudges. Moreover, some vegetables and fruits have become very expensive, so increasing opportunities to buy them might not help much if people are poor.

35 The case is, as we saw, different when we have to do with starvation, or a lack of certain key vitamins and minerals. But a healthy person temporarily deprived of food or water, and therefore experiencing “weakness,” or headache, also needs water and nutrition, and will recover quickly when provided with what their body needs. However, in such cases nudges seem to be of little use.

36 It is, once again, debatable whether one nudge really can create a habit, or whether the person after having been nudged once or twice will consciously decide to make the action a habit.

37 For example, not suffer from akrasia or be addicted, and for such reasons be unable to act on second-order preferences and choices.

38 Thus, I here take autonomy to be an internal feature of a person, and leave external freedom out of the definition. I, furthermore, believe that the above aspects of autonomy imply, at least, rationality and having acquired relevant knowledge. The above suggestion lies close to the three conceptions of autonomy suggested by Vugts et al. (2020, 115 ff.), namely, freedom of choice, agency, and self-constitution.

39 There even existed a lab at Stanford University called *Persuasion technology lab*, where the techniques behind these strategies were taught (Williams 2018).

40 No wonder, then, that young people are to an increasing extent experiencing “mental ill health” (UNICEF 2023). Reduced ability for autonomy most likely contributes to increases in existential anxiety, since such a reduction implies that some control over life (empowerment) is lost.

41 Not everyone holds this view, and some writers downplay such worries (see Kuyer & Gordijn, 2023, 215).

42 This critique and these worries are sometimes related to a concern about the emergence of a “nudging state,” with a new kind of questionable policy that searches for cost-effectiveness, quantifiable metrics, scalability, and interventions that can target heterogeneous populations (Button 2019, 1041).

43 One might also consider Buchanan’s closely related “education model,” which is encompassed by his broader “humanistic approach” (2023, 224 ff.).

44 However, we have already seen the importance of, and need for, opportunity creating policies.
